# Direct evidence for the role of microbial community composition in the formation of soil organic matter composition and persistence

**DOI:** 10.1038/s43705-021-00071-7

**Published:** 2021-11-06

**Authors:** Luiz A. Domeignoz-Horta, Melissa Shinfuku, Pilar Junier, Simon Poirier, Eric Verrecchia, David Sebag, Kristen M. DeAngelis

**Affiliations:** 1 Department of Microbiology, University of Massachusetts, Amherst, MA USA; 2 Department of Evolutionary Biology and Environmental Studies, University of Zurich, Zurich, Switzerland; 3 Laboratory of Microbiology, University of Neuchâtel, Neuchâtel, Switzerland; 4 IFP Energies Nouvelles, Rueil-Malmaison, France; 5 Institute of Earth Surface Dynamics, University of Lausanne, Lausanne, Switzerland

**Keywords:** Microbial ecology, Environmental microbiology

## Abstract

The largest terrestrial carbon sink on earth is soil carbon stocks. As the climate changes, the rate at which the Earth’s climate warms depends in part on the persistence of soil organic carbon. Microbial turnover forms the backbone of soil organic matter (SOM) formation and it has been recently proposed that SOM molecular complexity is a key driver of stability. Despite this, the links between microbial diversity, chemical complexity and biogeochemical nature of SOM remain missing. Here we tested the hypotheses that distinct microbial communities shape the composition of SOM, and microbial-derived SOM has distinct decomposition potential depending on its community of origin. We inoculated microbial communities of varying diversities into a model soil matrix amended with simple carbon (cellobiose) and measured the thermal stability of the resultant SOM. Using a Rock-Eval^®^ ramped thermal analysis, we found that microbial community composition drives the chemical fingerprint of soil carbon. While diversity was not a driver of SOM composition, bacteria-only communities lead to more thermally labile soil C pools than communities with bacteria and fungi. Our results provide direct evidence for a link between microbial community structure, SOM composition, and thermal stability. This evidence demonstrates the relevance of soil microorganisms in building persistent SOM stocks.

## Introduction

One of the grand challenge questions in microbiology is: when and where does “who’s there” matter for ecosystem functioning [1]? It has been postulated that diversity and microbial community structure matters for phylogenetically “narrow” processes such as denitrification [2–4], but not so much for phylogenetically-“broad” processes, such as carbon (C) cycling, which are completed by the majority of community members. However, recent work brings into question the assumption that all steps of C cycling are independent of “who’s there” [5, 6]. Moreover, community composition rather than diversity can have a wider impact on C cycling in soils [5–7]. Soil microbes are diverse in their macromolecular structures and metabolites [8] and therefore microbial-derived soil organic matter (SOM) may reflect distinctions across communities. SOM by its nature is molecularly diverse, and it was recently hypothesized that more diverse SOM persists longer in soil [9]. Here we provide empirical data to support the hypothesis that distinct communities inoculated into a model soil shape the composition of SOM and that this microbial-derived SOM has distinct decomposition potential depending on its community of origin.

## Results and discussion

**Fig. 1:**
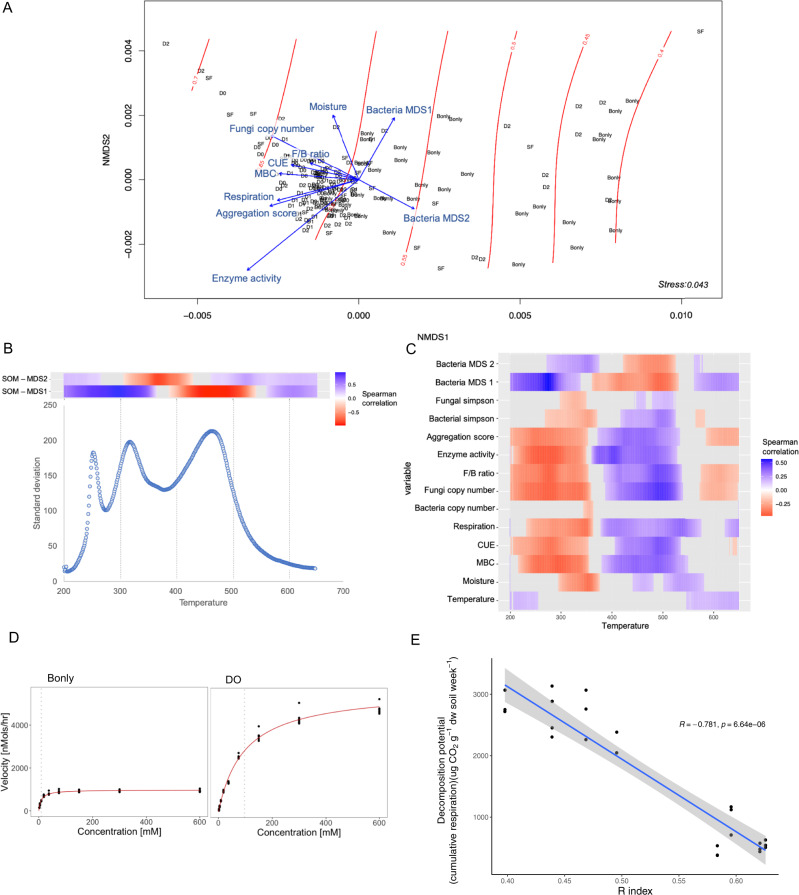
Drivers of SOM composition and persistence. Ordination of soil organic matter composition originated from a microbial diversity experiment in which a soil inoculum from a temperate forest was manipulated by consecutive dilutions (D0 > D1 > D2); selection of spore-forming microorganisms (SF); fungal exclusion (“B_only_”); inoculated into a model soil and grown on cellobiose as sole carbon source for 120 days under two temperatures (15 ^o^C and 25 ^o^C) and two moistures (30% and 60% WHC) in a full factorial design. Non-metric multidimensional scaling of Bray–Curtis distance from the pyrolyzed fraction of SOM based on Rock-Eval^®^ analysis. Red contour lines represent the SOM thermal-stability R-index with higher numbers indicating more thermal-stable SOM. Significant explanatory variables (*P* < 0.05) are represented as blue vectors and the lengths of the arrows are proportional to the strength of the correlation; enzyme activity corresponds to the maximum activity recorded (Vmax g^−1^ dw soil); Bacteria MDS1 and MDS2 represent the first and second axis of the bacterial community structure, respectively; Fungal copy number and bacterial copy number correspond to the quantification by qPCR of ITS and 16 S rRNA gene (copy number g^−1^ dw soil); F/B ratio correspond to the fungal to bacterial ratio abundance; CUE represents the carbon use efficiency; MBC corresponds to microbial biomass carbon (µg C g^−1^ dw soil); Respiration represents the cumulative respiration measured during microcosms incubation (C-CO_2_ g^-1^ dw soil) and aggregation score represents the water stable aggregate formation at the end of incubation (**A**). Spearman correlation between the SOM ordinations axes points and the FID signal captured at different temperatures and standard deviation of signal across all microcosms by temperature (**B**). Spearman correlation between biotic variables and abiotic experimental treatment conditions and the FID signal captured at each temperature (**C**). Betaglucosidase enzymatic kinetics at representative samples for “B_only_” and D0 treatments, vertical line represents the Km (**D**). The relationship between thermal-stability R-index and decomposition potential measured in a follow-up experiment in which soil generated during the 120 days of incubation was inoculated with another community and cumulative respiration measured as a proxy for decomposition potential of microbial-derived SOM (**E**).

Soil-derived microbial communities were subject to diversity removal by treatments with dilution (D0 > D1 > D2), filtering (bacteria predominantly “B_only_”), and heat (spore forming “SF”), and incubated under different moisture and temperature in order to generate distinct microbial communities in a model soil matrix [6]. In a sibling study aiming to disentangle the biotic and abiotic drivers of carbon use efficiency, we observed that the microbial community characteristics, e.g. bacterial community structure, bacterial diversity, fungi presence, and enzymatic activity influenced microbial community carbon use efficiency [6]. Here, we analyzed the formed SOM after four months of growth on cellobiose, using a method commonly used to quantify thermal stability and gradual stabilization of SOM [10]. The hydrocarbon compounds released at each temperature for each sample during the pyrolytic phase of Rock-Eval^®^ was used to calculate the Bray–Curtis-based chemical dissimilarity of the soil samples as a proxy for soil C composition, and the and the Rock-Eval^®^ thermal stability index (R-index) was calculated as a proxy for C persistence, as previously [10]. Bacterial or fungal diversity did not drive SOM composition. However, the resultant NMDS and analysis of similarity (ANOSIM) (R = 0.198, *P* < 0.0001) show that communities with distinct composition generated different SOM (Fig. 1A). The SOM fingerprint reflected the bacterial community composition (Fig. 1A, Procrustes statistics, cor = 0.2070, *P* = 0.0057) indicating that the bacterial community composition drove the formation of SOM composition. Moreover, the ordination first axis was strongly correlated with the Rock-Eval^®^ R-index (*ρ* = −0.95, *P* < 0.0001) which quantifies the relative contribution of thermally stable compounds [10] (i.e., compounds that require higher activation energy for thermal-decomposition). Thus, this suggests that distinct microbial communities produced SOM with different degrees of thermal stability.

Interestingly, the fungal community composition seemed to be less important in driving the SOM signature (Procrustes statistics; cor = 0.143, *P* = 0.0782). However, fungal abundance was positively related to the thermal stability of SOM (Fig. 1A and cor = 0.44, *t* = 6.4248, df = 168, *P* < 0.0001), supporting the role of fungi in overall community decomposition efficiency. This result agrees with research suggesting that fungi are major drivers of C cycling in soils [11]. Thus, while fungi were crucial for substrate decomposition, the SOM formed in these soils was a reflection of its bacterial community composition. Future studies can further elucidate if fungi and bacteria might play complementary roles during the decomposition of thermally-labile compounds and formation of more persistent SOM.

We next looked in more detail at the range of temperatures that captured the variation observed within the SOM ordination axes (Fig. 1B) and the biological community characteristics that drove the differences in the fingerprint of SOM (Fig. 1C). The signal captured across the temperature range reflected different aspects of the microbial community biological characteristics. While we previously reported that bacterial community diversity showed a positive saturating relationship with carbon use efficiency in these soils [6], our new results suggest a limited impact of diversity on driving SOM composition compared to microbial community structure and other community characteristics (Fig. 1A–C). For example, communities that grew more efficiently (i.e., high CUE) at the end of the incubation were also associated with more thermally stable SOM (Fig. 1A–C). This is consistent with the theory stipulating that high growth efficiency is ultimately associated with greater soil carbon retention [12–14]. Communities depleted of fungi were characterized by low growth efficiency and low biomass in these soils, and also had some of the most thermally labile SOM (Fig. 1A). Although low biomass was a predictor of the SOM signature (Fig. 1A–C), this was not due to residual added sugar. Indeed, the patterns of lower SOM thermal stability in lower diversity communities held even when the lowest temperature, predominantly sugar-rich peak [14] was removed and the statistical analysis repeated (Supplementary material). Therefore, community composition acts independently of efficiency and biomass to drive SOM composition.

Using variance partitioning we investigated the drivers of R-index (Supplementary material). Notably, microbial activity variables explained most of the R-index variance. This suggests that microbial activity and the by-products of their metabolism, such as extracellular enzymes, drove the formation of more thermally stable SOM. This agrees with the idea that microbial processing of C contributes to the formation of more persistent SOM pools [13].

Fungi and bacteria are considered to play different roles in soil C cycling [15, 16]. Accordingly, we observed distinct extracellular enzymatic dynamics in microcosms dominated by bacteria (“B_only_”) compared to microcosms with bacteria and fungi growing concomitantly (“D0”). B_only_ microcosms showed a reduced maximum enzymatic activity (Fig. 1D) (*V*_max_) (*P* < 0.0001, F = 16.43, df = 136) and Michaelis constant (*K*_m_) (*P* < 0.01, F = 5.195, df = 136) compared to treatments in which fungi were present, which should result in a smaller uptake of C and reduced microbial turnover of SOM [12, 15]. This could indicate that additional transformations of SOM occurred in the communities with bacteria and fungi compared to “bacteria-only” communities. These results highlight the potential loss in soil C cycling due to fungal exclusion and the relevance of fungi for soil functioning [6, 11, 15, 16]. Moreover, the bacterial communities may have benefitted from by-products of fungal growth and metabolism [16, 17]—leading to increasingly thermally stable SOM. While previous findings suggest that decomposition of fungal residues is an important regulator of C accumulation in soils [11], our results highlight the need of future studies elucidating if fungal ↔ bacterial interactions play an important role in this process.

Finally, to verify if more thermally stable SOM results in less available substrate to microorganisms, we conducted a follow-up experiment by inoculating a subset of microcosms from the first experiment with a diverse soil microbial inoculum similar to our D0 treatment and measured cumulative respiration as a proxy for potential decomposition of microbially-derived SOM. As we predicted, we observed a negative relationship between thermal stability and cumulative respiration (Fig. 1E). This suggests that more thermally stable C is less biodegradable and more likely to become part of more persistent soil–carbon stocks. Future studies should evaluate if this relationship changes under longer-time scales.

Model soils can be used to increase our understanding of major microbial ecology questions as it provides a single platform able to isolate specific components from confounding factors compared to natural soils [6, 18]. Here, by using a model soil, we show that microbial community composition and community characteristics drove the signature of the SOM and its thermal stability. Altogether, our results highlight the need for future studies investigating the role of fungal ↔ bacterial interactions for the decomposition efficiency and the formation of microbial-derived persistent SOM.

## Supplementary information

Supplementary Information

## Data Availability

The data and code supporting the findings presented here are available from the corresponding author on request and from Open Science Framework Repository project: https://osf.io/evb6d/.
