# Supplementary material for: Direct evidence for the role of microbial community composition in the formation of soil organic matter composition and persistence
Source: ISME Commun. 2021 Nov 6;1:64. doi: 10.1038/s43705-021-00071-7 (PMC9723721; doi:10.1038/s43705-021-00071-7)
Supplement: Supplementary file 1 — Supplementary Information [file 43705_2021_71_MOESM1_ESM.docx]

**Supplementary Materials**

**Materials and Methods**

**Data collection**

Microbial communities of varying complexities resulting from diluting and filtering soil were inoculated into a model soil matrix and amended with cellobiose and ammonium nitrate on a weekly basis^6^. At the end of four months of incubation, soil was characterized for respiration, growth, microbial biomass carbon, water-stable aggregate formation, betaglucosidase extracellular enzyme activity, and the bacterial and fungal communities were sequenced as described in Domeignoz-Horta et al. 2020^6^. To characterize the microbial communities we used the microbial community ordinations axes (MDS Axis 1 and MDS axis 2) for bacterial and fungal communities, and the simpson diversity index for both bacteria and fungi was calculated. The abundance of fungal and bacterial cells was determined by gene copy number of a phylogenetic marker gene as described in Domeignoz-Horta et al. 2020^6^. Microbial communities were maintained under sterile conditions during incubation and negative controls (i.e. substrate added without inoculum) allow us to verify that we got no contamination during the incubation. The sequencing results confirmed that D0 had higher diversity at the end of incubation with an average of 50.1 (CI_95%_ = 45.4 – 54.7) bacterial OTUs and 39.9 (CI_95%_ = 36.8 – 43.0) fungal OTUs compared to D1 with 11.5 (CI_95%_ = 9.9 – 13.1) for bacteria and 16.8 (CI_95%_ = 14.6 – 18.9) for fungi and D2 with 9.7 (CI_95%_ = 8.3 – 11.2) for bacteria and 22.4 (CI_95%_ = 16.4 – 28.4) for fungi, respectively. Air-dried soil was subject to Rock-Eval^®^ ramped thermal pyrolysis following the protocol of Sebag et al. 2016^10^. To examine the relationship between SOM thermal-stability and its bio-availability to microorganisms, we performed a follow up experiment in which we inoculated a subset of soils from the previous experiment with a natural soil-derived microbial inoculum. These microcosms were maintained at 60% water holding capacity and we monitored respiration daily during one week as a proxy for bio-available decomposable C.

**Data Analysis**

Statistical analyses were performed in R statistical software (version 3.6.3), using the vegan and agricolae packages. For Rock-Eval^®^ analysis, soil samples are pyrolyzed from 200 to 650^o^C, followed by combustion of residual C from 400 to 850^o^C. Hydrocarbon compounds (HC) released during this process are measured through time by a flame ionization detector (FID) and generate a HC thermogram for each sample. Bray-Curtis distance of thermograms was calculated using mean C released at each 1^o^C unit of temperature and used as input to a non-metric multidimensional scaling (NMDS) to describe microcosms C signature. The variables that significantly explained the C signature amongst the different microcosms were identified using the *envfit* function (permutation tests n = 10000, *P* < 0.05). Surface fitting of the Rock-Eval^®^ R-index within the ordination was performed using the *ordisurf* function. Here we used the thermogram generated during pyrolysis as a proxy for SOM chemical composition. Procrustes analysis was used to test if the distance matrix of bacterial and fungal communities show superposition with the distance matrix of SOM (permutations test n = 10000, *P* < 0.05). We used Spearman correlations to explore relationships between C released at specific temperatures and the overall SOM ordination and biotic variables. We calculated the SOM thermal stability R-index as previously^10^.

Supplementary Text 1.

Variation partitioning analysis was applied to disentangle the contribution of biotic drivers and abiotic factors to the thermal stability R-index using the varpart function in the vegan package. Briefly, significant explanatory variables were selected using a backwards stepwise model selection process and an RDA (*P* < 0.05). Variables were then grouped into one of the following categories: microbial community diversity/structure, microbial activity, microbial abundance, or abiotic. All factors jointly explained 65% of the R-index variance, with activity being the strongest predictor contributing alone with 15%of the variance.

To verify that lower SOM signature was not driven by residual substrate we repeated our SOM ordination analysis excluding the signal captured in the first range of temperatures (200-275^o^C) as this is predominantly driven by a sugar-rich peak. This analysis showed very similar results to our analysis with the full dataset as the following variables significantly explained SOM ordination: moisture (r^2^ = 0.085, *P* = 0.0010), microbial biomass carbon (r^2^ = 0.12, *P* = 0.0002), carbon use efficiency (r^2^ = 0.087, *P* = 0.0014), bacteria MDS axis 1 (r^2^ = 0.095, *P* = 0.0005), bacteria MDS axis 2 (r^2^ = 0.0699, *P* = .0055), fungal:bacterial ratio (r^2^ = 0.048, *P* = 0.0457), aggregation score (r^2^ = 0.151, *P* < 0.0001), cumulative respiration (r^2^ = 0.151, *P* = 0.0001), ITS copy number (r^2^ = 0.187, *P* < 0.0001) and extracellular enzyme activity (Vmax) (r^2^ = 0.419, *P* < 0.0001).
